# Balloon sinuplasty in patient with cerebral palsy: result and follow-up

**DOI:** 10.1590/S1808-86942010000300024

**Published:** 2015-10-20

**Authors:** João Flávio Nogueira Júnior, Maria Laura Solferini Silva, Aldo Cassol Stamm

**Affiliations:** 1 Specialist in otorhinolaryngology, ABORL-CCF; otorhinolaryngologist at the Sao Paulo Otorhinolaryngology Center, Prof. Edmundo Vasconcelos Hospital; 2 Medical resident in otorhinolaryngology, Sao Paulo Otorhinolaryngology Center, Prof. Edmundo Vasconcelos Hospital; 3 Doctoral degree, director of the Sao Paulo Otorhinolaryngology Center, Prof. Edmundo Vasconcelos Hospital. Sao Paulo Otorhinolaryngology Center, Prof. Edmundo Vasconcelos Hospital Rua Borges Lagoa 1450 3o. Andar do Prédio dos Ambulatórios – São Paulo SP.

**Keywords:** video-assisted surgery, postoperative care, postoperative period, surgical procedures, sinusitis

## INTRODUCTION

Dilatation of paranasal sinus ostia or sinuplasty has yielded interesting results in the treatment of chronic rhinosinusitis.^1^,^2^Sinuplasty applied the concept of remodeling of these structures without removal of bone or mucosa.^1^, ^2^, ^3^

Preserving these tissues may be an advantage of this procedure, as complications such as decreased mucociliary movements, scarring, synechiae, and postoperative bleeding occur less frequently in patients that undergo dilatation.^1^,^2^,^4^

Postoperative care is also simpler; as tissue is not removed and the nasal mucosa is not traumatized, there is less formation of crusts and there is less need of nasal dressings. This may be interesting especially in patients that find it difficult to perform postoperative cleaning, such as children and patients with handicaps such as cerebral palsy.^3^,^4^

We present the case of a youth with cerebral palsy and chronic rhinosinusitis that underwent sinuplasty, and discuss the postoperative follow-up.

## CASE REPORT

ASPN, a male 18-year-old patient with cerebral palsy presented with a 5-year diagnosis of chronic rhinosinusitis, for which he had been treated unsuccessfully with medication. Two months ago he presented orbitary cellulitis to the right and meningitis as complications of frontal and ethmoid sinusitis. The patient was admitted to hospital and successfully treated with medication.

The patient was forwarded to our unit; computed tomography showed signs of right frontal rhinosinustis.

After clarifications, consent from caretakers, and preoperative work-up, the patient underwent right frontal sinus sinuplasty.

Surgery was performed under general anesthesia. The Relieva Sinus Balloon Catheter System (Acclarent, United States) was used with a transillumination technique.^4^

**Figure 1:**
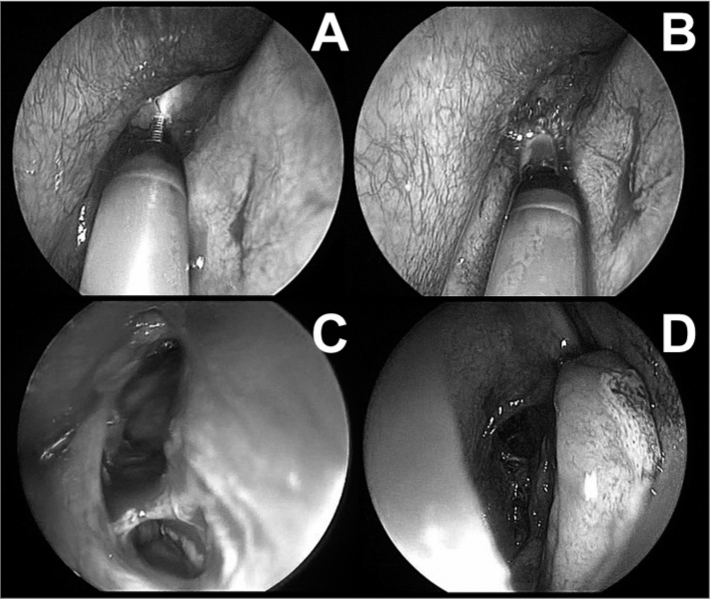
Right frontal sinus sinuplasty. A: endoscopic view (45 degrees, 4mm) of the right middle meatus and placement of a guide wire in the right frontal sinus under transillumination. B: endoscopic view (45 degrees, 4 mm) of balloon placement for dilating the right frontal sinus recess and ostium. C: endoscopic view (45 degrees, 4mm) after dilatation and lavage of the right frontal sinus. D: endoscopic view (0 degrees, 4mm) of the right middle meatus after sinuplasty. Note almost complete absence of nasal mucosa trauma.

A 5mm balloon was introduced and dilated up to 12 atmospheres of pressure with a saline solution at several points along the right frontal recess (Figure 1). Next, a lavage catheter was introduced to clean the secretions.

The procedure lasted about 60 minutes. No nasal packing was applied. There was no bleeding or any other complication.

The patient returned on the postoperative days 7, 14, 30 and 60 days. Superficial cleaning was carried out, as the patient did not allow cleaning with endoscopy. A second computed tomography was done of the 60th postoperative day; it showed normally aerated paranasal cavities.

## DISCUSSION

Management of chronic rhinosinusitis is a challenge for otorhinolaryngologists in some patients.^5^ Tiered therapy is applied, but when unsuccessful, nasal endoscopic surgery is indicated.^5^

Nasal endoscopic surgery is safe and yields excellent results; however, inherent complications may occur, such as synechiae, crusts, and epistaxis, especially in special patient groups who often do not carry out appropriate postoperative care.^2^,^5^

A minimally invasive technique has recently been introduced in our country, namely balloon sinuplasty. This procedure is safe and so far has yielded good results.^2^,^3^

Sinuplasty appears to offer advantages compared to traditional nasal endoscopic surgery, such as decreased surgical time, shorter hospital stays, fewer complications (bleeding and synechiae), and more important, less need for postoperative care.^2^,^3^,^4^

This tool is limited in that it cannot adequately cauterize and dilate a specific sinus where the anatomy is not favorable;1 some authors have questioned whether dilatation yields only temporary results.6 Complications are few when using this method; these may include cerebrospinal fluid leaks, as with nasal endoscopic surgery.^1^,^3^,^4^

## FINAL COMMENTS

There were no technical difficulties or complications of sinuplasty in this patient. After 60 days the youth reports that symptoms have regressed; additionally, computed tomography images of the procedure have normalized.

Sinuplasty may be an alternative to surgery in some patients; it is a minimally invasive procedure that preserves the nasal mucosa and paranasal sinus ostia. Case series with longer follow-up periods are needed.
